# Inclusive fitness and differential productivity across the life course determine intergenerational transfers in a small-scale human society

**DOI:** 10.1098/rspb.2014.2808

**Published:** 2015-03-22

**Authors:** Paul L. Hooper, Michael Gurven, Jeffrey Winking, Hillard S. Kaplan

**Affiliations:** 1 Department of Anthropology, Emory University, 1557 Dickey Drive, Atlanta, GA 30322, USA; 2 Santa Fe Institute, 1399 Hyde Park Road, Santa Fe, NM 87501, USA; 3 Department of Anthropology, University of California Santa Barbara, Santa Barbara, CA 93106, USA; 4 Department of Anthropology, Texas A&M University, Mailstop 4352, College Station, TX 77843, USA; 5 Department of Anthropology, University of New Mexico, MSC01–1040, Albuquerque, NM 87131, USA

**Keywords:** human life history, inclusive fitness, intergenerational transfers, parental investment, grandparental investment, food sharing

## Abstract

Transfers of resources between generations are an essential element in current models of human life-history evolution accounting for prolonged development, extended lifespan and menopause. Integrating these models with Hamilton's theory of inclusive fitness, we predict that the interaction of biological kinship with the age-schedule of resource production should be a key driver of intergenerational transfers. In the empirical case of Tsimane’ forager–horticulturalists in Bolivian Amazonia, we provide a detailed characterization of net transfers of food according to age, sex, kinship and the net need of donors and recipients. We show that parents, grandparents and siblings provide significant net downward transfers of food across generations. We demonstrate that the extent of provisioning responds facultatively to variation in the productivity and demographic composition of families, as predicted by the theory. We hypothesize that the motivation to provide these critical transfers is a fundamental force that binds together human nuclear and extended families. The ubiquity of three-generational families in human societies may thus be a direct reflection of fundamental evolutionary constraints on an organism's life-history and social organization.

## 1. Introduction

Relative to other primates and mammals, humans show remarkably late maturation, extended lifespan and reproductive cessation preceding general somatic senescence [1–3]. Recent theories of human life-history evolution have indicated a crucial role for intergenerational resource transfers in supporting these distinctive features of human life-history [4–7]. These theories propose that transfers increasing the fertility and survival of close relatives constitute a form of ‘indirect’ reproduction, which has allowed selection to favour the evolution of significant post-reproductive lifespan. While a number of empirical studies have provided hints regarding the structure of fitness-enhancing transfers [7–10], few have provided detailed statistical breakdowns of their direction and volume across the life course.

In terms of theory, existing models of intergenerational transfers have not fully integrated the inclusive fitness motivations for transfers with the economics of resource production and consumption across the life course. Rogers' model of the evolution of menopause [11] considered direct demographic effects of kin altruism within the framework of inclusive fitness theory [12] but did not explicitly treat economic production or transfers. The life-history models of Kaplan & Robson [4,13,14] and Lee [6,15], on the other hand, represented transfers by allowing costless borrowing and lending of resources across different ages within lineages, and thus effectively assumed perfect relatedness between donors and recipients.

Extending the theory of intergenerational transfers to capture the reality of imperfect and variable relatedness between individuals has three advantages. First, it considers the effects of transfers on the long-term inclusive fitness of donors, and gives a basis for their evolutionarily stability in the face of selection. Second, by specifying who is expected to provide net transfers to whom, under what circumstances, it provides predictions for the heterogeneous structure of networks of social support and investment, and moves away from unrealistic assumptions of homogeneous interactions within groups. Third, the analysis enriches inclusive fitness theory by providing a concrete and systematic source of variation in the benefits and costs of kin altruism, which is based on the life-history of development and productivity, rather than focusing on genetic relatedness alone [16].

This paper has three related goals. The first is to integrate Hamilton's theory of inclusive fitness with life-history theory and derive predictions for the relationship between production, consumption, kinship and transfers. The second is to empirically characterize net caloric production and transfers, as they vary by sex, age, productivity and relationship, in one small-scale subsistence-based human society, the Tsimane’ of lowland Bolivia. The third is to test the predictions derived from the theory and assess the extent to which observed patterns of transfers are explained by kin selection and life-history theory.

The following section develops the theory of transfers on the basis of kin selection and life-history and derives predictions for expected patterns of resource flows across life in human economic systems. This is followed by an empirical analysis of food transfers within and between families from a study of 1254 individuals in 8 Tsimane’ communities over a mean period of 14 months. The findings are then discussed in the light of the theory and the evolution of human and non-human life histories more generally.

## 2. Theory and predictions

Hamilton's theory of inclusive fitness predicts that selection will favour kinship-based altruism when the benefit of the altruistic act to the recipient (*b*) devalued by the coefficient of relatedness between the two individuals (*r*) exceeds the cost to the donor (*c*), or *br* > *c* [12]. Life-history theory provides a specific means of understanding how both the benefits and costs of altruistic transfers are likely to vary systematically across the life course.

For a given pair of individuals or families, the relationship between age and the marginal benefit of receiving calories, on the one hand, and between age and the marginal cost of giving calories, on the other, should depend on both the availability of calories and the benefits of consumption at that age. These benefits and costs are fundamentally determined by the age-schedules of productive ability and work. For organisms that require learning to achieve adult competence, early in life—when individuals are relatively inefficient producers and cannot meet their energy requirements through their own work effort—the marginal benefit of receiving calories from others should be relatively higher. Conversely, when efficiency is high at older ages, the marginal cost of giving away calories should be reduced, owing to high productivity and diminishing returns to personal consumption.

**Figure 1.**
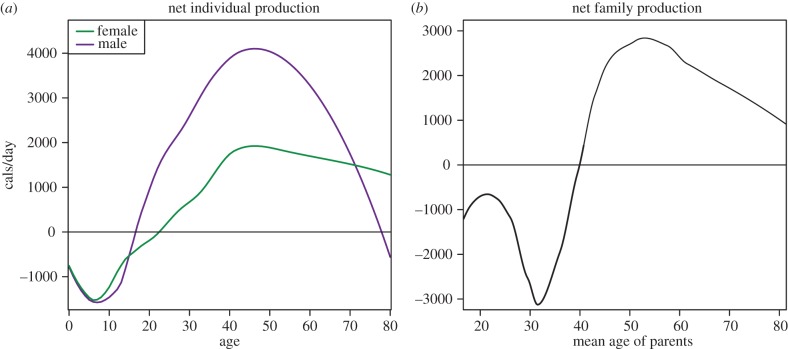
Net production of Tsimane’ (*a*) individuals and (*b*) nuclear families.

Data from small-scale human societies show that the asymmetry between low early-life productivity and high later-life productivity is especially great for humans compared to chimpanzees [8,9,17–20]. The life-history of net caloric productivity (gross daily production minus consumption) in the Tsimane’ case is given in figure 1*a*. On average, Tsimane’ offspring consume more than they produce for most of the first two decades of life. Later in life, from the late 20s through the 60s, adults produce major caloric surpluses that far outstrip individual consumption requirements. Such large asymmetries in the ability to produce calories across life are predicted to generate differential costs of giving, and benefits of receiving, that motivate substantial net transfers from older to younger kin within nuclear and extended families.

Among the Tsimane’, asymmetries in individual productivity and consumption also give rise to systematic imbalances in the caloric budgets of nuclear families (single or married adults and their immediate dependents, who live and eat together as a unit), as shown in figure 1*b*. On average, younger families—with parents not yet at peak productivity, and multiple unproductive dependent offspring—show net caloric deficits; while older families—with highly productive parents, and few young dependents—produce net surpluses. The theoretical implication of this is that nuclear families with more dependents and/or lower productivity should face higher gains from receiving and higher costs of giving; while those with fewer dependents and/or higher productivity should face lower gains from receiving and lower costs of giving.

This study provides a number of explicit tests of the theory developed here and in preceding work [5,7,8,21]. First, in an analysis of transfers between individuals, we test the fundamental predictions **P1** and **P2**, that parents and grandparents provide significant net downward transfers to offspring and grandoffspring, respectively. Second, we leverage variation in the caloric budgets of nuclear families (i.e. the extent of net caloric surplus or deficit) to test predictions arising from the application of inclusive fitness theory to economic life-history. The multiplicative role of *r* in Hamilton's rule (*br* > *c* or, equivalently, *b* > *c*/*r*) implies that need and relatedness *interact* in the determination of transfers. In other words, need should only be associated with net transfers among related individuals, and more so as relatedness increases. **P3** therefore predicts that relatedness interacts *negatively* with the net need of donors, whereas **P4** predicts that relatedness interacts *positively* with the net need of recipients in determining net transfers.

The current theory does not predict that net need, independent of relatedness, should be associated with substantial net transfers. It is important to note, however, that non-kin should be expected to benefit from exchanging food reciprocally when differing in relative need over short time-scales, an observation that has been well attested in many analyses of food sharing in small-scale societies [22–24]. The current study, however, aims to examine the effect of long-term net need based on the demographic composition of families, rather than fluctuations over shorter time-scales of weeks and months. Thus, with the present operationalization, if exchange relationships are—on average—reciprocally balanced across the time period examined, we would still expect more-or-less zero net transfers between more distant kin and non-kin pairs.

Prior studies of resource transfers in small-scale human societies have often focused on direct associations between kinship and transfers [25–30], without treating the moderating effects of life-history stage. Several studies have considered the effect of reproductive value on sharing [31,32], which tends to produce downward transfers given competing recipients with the same *r*. Others have evaluated the relationship between transfers and indices of familial need (e.g. number of dependents or producer : consumer ratios) without explicitly considering their interaction with kinship [25,28,29,31]. In a study of Ache reservation food-sharing prefiguring the current approach, Allen-Arave *et al.* [33] examined the relationship between net transfers and the interaction of *r* with the difference in the net need of households, but did not separate the effects of donor versus recipient need, nor characterize transfers at the individual level.

The current study—based on *b* and *c* in Hamilton's equation, as determined by the constraints of life-history, productivity and individual differences—complements these past approaches. Here, we empirically operationalize variation in *b* and *c* across life; predict the direction and volume of net transfers between individuals and families on the basis of these variables; then test the predictions in a series of statistical models. In combination with kinship, the index of net need used in this study—daily consumption minus gross production, estimated from high-resolution individual-level data over a more than one-year study period—provides a continuous predictor for the strength of relationships across Tsimane’ extended families.

## 3. Material and methods

### (a) Data collection

Data were collected through fieldwork with Tsimane’ forager–horticulturalists between 2005 and 2010 under the aegis of the Tsimane’ Health and Life History Project [34,35]. The Tsimane’ are an Amerindian group native to the Beni Department of lowland Bolivia [36–39]. Production-and-sharing interviews covering subsistence economic activities were conducted with families in the Tsimane’ language roughly twice per week. The production activities and returns of each family member in the preceding two days were queried and recorded. For each food product produced, interviewees were asked which individuals had consumed portions of the product in prepared meals, and which had received portions of the product as raw gifts, in what quantity. Additionally, horticultural field interviews were conducted with each family on a yearly basis that documented labour contributions to fields and final crop yields. Further details on the interview sample, methods and the calculation of daily food production, consumption, transfers and kinship are given in the electronic supplementary material, S1 and [35].

### (b) Statistical analysis

Three sets of models were estimated to evaluate the direction, volume and statistical significance of net transfers within communities: (set A) net transfers *from focal individuals* to all children, grandchildren, spouses and children-in-law (electronic supplementary material, tables E1, E3, E5 and E6); (set B) net transfers *to focal individuals* from all parents, grandparents and parents-in-law (electronic supplementary material, tables E2, E4 and E7); and (set C) net transfers *between nuclear families* (tables 1 and 2).

**Table 1.** Mixed-effect models predicting net transfers (calories per day) from older nuclear family *i* to younger nuclear family *j*, as a function of genetic relatedness, net caloric need and their interaction. Net transfers and need are standardized to have mean = 0 and s.d. = 1. *n* = 3279 family–family dyads. Further details on the models and variables are given in the electronic supplementary material, S2.2. *p*-values indicate whether the regression coefficient B deviates significantly from the null expectation of zero.

predictors of net transfer fam. *i* → fam. *j*	1. estimated net need model			2. measured net need model		
	B	s.e.	*p*-value	B	s.e.	*p*-value
intercept	0.013	0.146	0.121	0.011	0.146	0.137
*r*	1.010	0.278	0.001	1.135	0.271	<0.001
net need of *i*	−0.001	0.020	0.497	−0.019	0.021	0.123
net need of *j*	0.010	0.020	0.306	0.019	0.021	0.172
net need of *i* × *r*	−1.465	0.298	<0.001	−1.656	0.268	0.001
net need of *j* × *r*	0.607	0.250	0.010	0.774	0.221	0.001

**Table 2.** Mixed-effect models predicting net transfers (calories per day) from older nuclear family *i* to younger nuclear family *j*, as a function of familial net need for each relationship category. Model 1 reports the mean net transfer within each relationship type. Models 2 and 3 report the standardized regression coefficient *β* for the relationship between net need and net transfers for donors and receivers within each relationship category. *p*-values indicate whether the mean or standardized regression coefficient *β* deviates significantly from the null expectation of zero.

					2. relationship between estimated net need and net transfer						3. relationship between measured net need and net transfer					
		1. net transfer			net need of *i*			net need of *j*			net need of *i*			net need of *j*		
family dyad relationship category	*n*	mean	s.e.	*p*-value	*β*	s.e.	*p*-value	*β*	s.e.	*p*-value	*β*	s.e.	*p*-value	*β*	s.e.	*p*-value
Par. *i* → child *j*	157	150.3	94.3	0.011	−0.643	0.088	<0.001	0.125	0.094	0.068	−0.962	0.082	<0.001	0.422	0.075	0.002
Sib. *i* → sib. *j*	225	113.4	91.6	0.023	−0.172	0.070	0.011	0.022	0.092	0.377	−0.166	0.067	0.016	0.070	0.062	0.074
other kin *i* → *j*	782	47.3	87.4	0.103	−0.017	0.036	0.295	0.026	0.057	0.292	0.000	0.030	0.523	0.013	0.053	0.428
non-kin *i* → *j*	2115	49.0	86.1	0.019	−0.006	0.022	0.391	0.037	0.027	0.077	−0.051	0.021	0.021	0.037	0.027	0.066

For the individual-level models in sets A and B, mixed-effect regression [40,41] was employed to characterize the sum of net food transfers between focal individuals and different categories of kin as a function of sex and age. For the family-level models in set C, mixed-effect regression was used to estimate net transfers from an older nuclear family *i* to a younger family *j* co-resident in the same community. These family-level models—which predict net transfers as a function of kinship, the net need of each family and their interaction—capture important patterns of secondary redistribution that occur with regular pooling of food within nuclear families (reproductive-age single or married adults and their immediate dependents). Two variables representing the net need of families—measured net need and an instrumental variable of estimated net need—were used in the analyses reported in tables 1 and 2; the interpretation of these variables is discussed in the electronic supplementary material, S2.2 and in §4.

## 4. Results

### (a) Transfers between individuals

**Figure 2.**
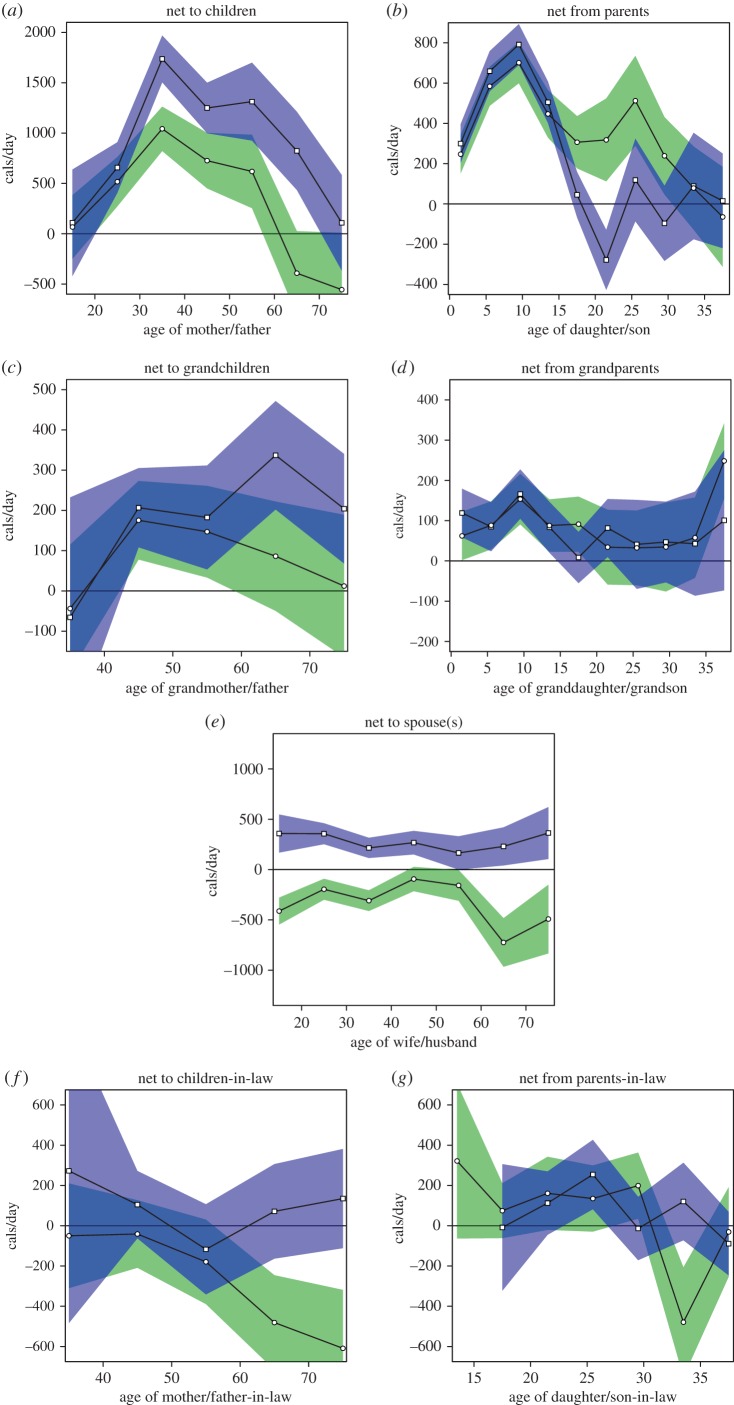
Net transfers of food as a function of donor and recipient age and sex. Means and standard errors are showed in green for females and purple for males. (*a*) Net from a focal mother/father to all children. (*b*) Net to a focal daughter/son from all parents. (*c*) Net from a focal grandmother/grandfather to all grandchildren. (*d*) Net to a focal granddaughter/grandson from all grandparents. (*e*) Net from a focal wife/husband to her/his spouse (or spouses). (*f*) Net from a focal mother/father-in-law to all children-in-law. (*g*) Net to a focal daughter/son-in-law from all parents-in-law. Values are derived from electronic supplementary material, tables E1–E7. Note that the *y*-axis varies depending on the scale of the net transfer values, and that transfers from mothers to offspring do not include contributions through lactation.

Figure 2 summarizes net transfers of food between generations within Tsimane’ extended families, based on model estimates reported in electronic supplementary material, tables E1–E7.

Mean net caloric transfers of primary production to all offspring from Tsimane’ fathers and mothers (above and beyond lactation) are given in figure 2*a* (electronic supplementary material, table E1). In support of **P1**, adults of both sexes contribute significantly positive net transfers of subsistence production to their offspring.

Downward net transfers of food from mothers to offspring are significantly positive from the 20s through the 50s, with an all-ages mean of 177 cals d^–1^ and a peak at 1042 cals d^–1^ during the 30s. On average, there is a tendency for mothers to become net recipients from their children beginning in their 60s (this relationship is not significant in the 60s, but significant at *p* = 0.03 in the 70s). Net downward transfers from fathers to their offspring are significant from the 20s through the 60s, with an all-ages mean of 1107 cals d^–1^ and a peak of 1737 cals d^–1^ during the 30s. Mean net transfers from fathers are positive but statistically indistinguishable from zero (*p* = 0.4) in the 70s.

Net transfers of subsistence production from an offspring's parents as a function of offspring age and sex are given in figure 2*b* (electronic supplementary material, table E2). Daughters receive significant net transfers from their parents into the second half of their 20s, with a mean of 437 cals d^–1^ net received, and a peak of 701 cals d^–1^ around age 10. Net transfers between daughters and their parents do not deviate significantly from zero from the mid-30s onward. Sons receive significant net transfers until about age 15, with a mean of 393 cals d^–1^ received and a peak of 792 cals d^–1^ around age 10. Young men in their early 20s are estimated to provide net upward transfers to their parents at a mean rate of 278 cals d^–1^ (*p* = 0.03). Mean net transfers between sons and parents are not significantly different from zero from the mid-20s onward.

The results in figure 2*c* (electronic supplementary material, table E3) support **P2**, that both Tsimane’ grandmothers and grandfathers provide significant net transfers to their grandchildren. Grandmothers are estimated to provide a significant mean net of 117 cals d^–1^ to their grandchildren (*p* = 0.002), with a peak of 176 cals d^–1^ during their 40s. Net transfers to grandchildren are significantly positive from grandmothers in their 40s and 50s; in the 60s and 70s, they are positive, but not statistically significant. Net transfers from grandfathers to their grandchildren average 223 cals d^–1^, with a peak of 337 cals d^–1^ in the 60s. Net transfers from a grandfather to his grandchildren are significantly positive from the 40s through the 70s.

Tsimane granddaughters and grandsons are estimated to receive significant net transfers from their grandparents from birth until roughly age 19 and 15, respectively (figure 2*d*; electronic supplementary material, table E4). Peak net transfers to grandchildren in the first two decades of life occur around age 10 in both sexes, at around 170 cals d^–1^. Net transfers to grandchildren are greater when the parents are not alive and co-resident in the same community: grandchildren under 12 receive a net of 90 more calories per day (*p* < 0.01) from their grandparents if their mother is absent, and 60 more calories per day (*p* = 0.03) if their father is absent. The significant net transfers to grandchildren in figure 2*d*, however, are only partly explained by compensation for parental absence, as grandchildren under 12 still receive an average of 164 cals d^–1^ (*p* < 0.001) from grandparents when both parents are present.

Figure 2*e* (electronic supplementary material, table E5) shows that Tsimane husbands provide significant net transfers of food energy to their wives, with a mean net of 273 cals d^–1^ (*p* < 0.001). Net transfers are consistently positive from husbands to wives across life, and significantly positive in more than half of age categories. Flows between husbands and wives are somewhat more even for wives in their 40s and 50s and husbands in their 50s and 60s, with a mean husband → wife net flow of 146 cals d^–1^ (0.12 > *p* > 0.23) during this period.

The net result of exchanges between Tsimane adults and their parents-in-law (i.e. their spouse's parents; figure 2*f*; electronic supplementary material, table E6) are less clearly patterned. Mean net transfers between a mother-in-law and her children's spouses have a tendency to flow upward (mean = 206 cals d^–1^), an effect that is significant in the all-ages model (*p* = 0.01), but not in the age-stratified model (*p* > 0.05). Mean transfers between fathers-in-law and their children's spouses have a slight but not significant (*p* > 0.1) tendency to occur downward, with a mean net of 67 cals d^–1^. In figure 2*g* (electronic supplementary material, table E7), daughters- and sons-in-law show a slight tendency to be net receivers from their parents-in-law (83 cals d^–1^ to daughters-in-law, *p* = 0.07, and 69 cals d^–1^ to sons-in-law, *p* = 0.09), although net flows from focal children-in-law are estimated to be upward for some age–sex categories (for example, to parents-in-law from daughters-in-law in their early 30s, *p* = 0.04).

Different results across sex, age and relationship category provide distinct angles on an individual's net contribution to her/his family across life. For example, mothers in their later 60s and 70s are estimated to receive net transfers from their adult children (474 cals d^–1^), while also providing net contributions of around 50 cals d^–1^ to their grandchildren. Sons in their early 20s who provide a direct net of 278 cals d^–1^ to their parents also receive 82 cals d^–1^ from their grandparents. While mothers-in-law tend to be net receivers on an individual-to-individual basis, children-in-law tend to be net receivers from parents-in-law when the contributions of fathers- and mothers-in-law are considered together.

### (b) Transfers between nuclear families

Table 1 presents the analysis of food sharing between Tsimane’ nuclear families based on mean genetic relatedness and net caloric need. The results show first, that relatedness is a robust independent predictor of net transfers from older to younger families; and second, that the effects of familial net need are consistent with the theoretical predictions.

In support of **P3**, there is a significant negative relationship between net transfers from family *i* to family *j*, and the interaction between family *i*'s net need and relatedness. Thus, the higher the productivity and the lower the consumption requirements of *i*, the greater the net transfer from *i* to *j*; the size of this effect, moreover, increases with increasing relatedness. Supporting **P4**, the relationship between net transfers and the interaction between family *j*'s net need and relatedness is significantly positive. Thus, the lower the productivity and the higher the consumption requirements of *j*, the greater the net transfer to *j*, an effect that increases with increasing relatedness.

The results in table 1 show that among closely related families, holding need constant, on average older families make net transfers to younger families. These net transfers from older to younger increase with the number of unproductive mouths in the younger household, but decrease with those in the older household. For the minority of closely related dyads in which the older family has more unproductive consumers than the younger, positive net flows are predicted to occur in the opposite direction, with the younger family providing net transfers to the older; in either case, energy flows to the demographically needier of the two families.

In table 1, the association of need with transfers, independent of relatedness, is mildly in the direction of net flows towards greater need (i.e. negative for the need of *i* and positive for the need of *j*). This is somewhat more pronounced when using the measured net need variable. These effects, however, are not statistically significant (*p* > 0.12). This suggests that neither donor nor recipient need alone is sufficient to produce substantial net transfers in the Tsimane sample; instead, transfers are predominantly directed from less needy to more needy kin according to the degree of kinship.

Table 2 breaks down the between-family analysis into four categories of relationship: parent–offspring pairs, sibling–sibling pairs, all other kin pairs (dyads with *r* > 0 other than parent–offspring and siblings) and non-kin pairs (*r* = 0). The analysis shows that the largest net transfers and effects of need occur between the families of parents and their adult offspring. These results reflect net investments in adult offspring and grandoffspring, and the responsiveness of these investments to both offspring/grandoffspring need and parental/grandparental surplus. Table 2 also indicates that significant net transfers occur from older to younger siblings and their families, including nieces and nephews. The effects of net need on transfers between the families of adult siblings are statistically significant for donor but not recipient need (i.e. for differences in *c* but not *b*).

Table 2 shows little to no net intergenerational transfers (or effects of need) between more distantly related nuclear families. Between non-kin, there is some evidence of mean net downward flows from older to younger families, and some responsiveness to need, particularly when using the measured net need of families (*p* ≥ 0.02). The effect sizes, however, are small (*β* < 0.06), and not statistically significant when employing the instrumental estimated net need variable.

In tables 1 and 2, it is reassuring that both the estimated and measured net need variables produce similar parameter estimates, suggesting that the main results are robust to endogeneity and measurement error (see the electronic supplementary material, S2.2). In both tables, the estimated effect sizes tend to be greater for measured need than estimated need. The interpretation of these differences is difficult; on the one hand, they may indicate that net transfers are sensitive to true heterogeneity in household productivity (above and beyond that associated with age and demographic composition), or medium-term variability in fortune captured during the sampling window; on the other hand, they may simply result from the endogenous, structural relationship between measured net productivity and net transfers discussed in electronic supplementary material, S2.2.

## 5. Discussion

This study shows that kinship and life-history jointly predict patterns of intergenerational investment in a small-scale human subsistence economy. The results show that Tsimane’ parents and grandparents are economically productive and provide net economic contributions to kin into the seventh decade of life. Between households, the net transfer of resources is predicted by the interaction of inclusive fitness interests with differential productivity and need. Households with higher productivity and fewer dependents provide net transfers to closely related, usually younger, households with lower productivity and more dependents.

These results are important, given that downward flows between generations could happen without respect to relatedness, and that kin selection alone does not dictate why flows should be downward. This is the first paper to fully unite the time-path of production with inclusive fitness theory and test the unified model in an empirical analysis. The data indicate that flows across Tsimane’ networks are exquisitely patterned, more so than either theory alone would predict.

This analysis supports life-history models suggesting that human demographic characteristics—long lifespan, slow development, high fertility and menopause—and intergenerational transfers have co-evolved in the history of our lineage [4–7,42,43]. Models emphasizing learning and embodied capital hypothesize that the movement of hominins into a production niche favouring early-life investments in brain and skill development was a primary driver behind the distinctiveness of the human life-history, including the extent and duration of intergenerational provisioning [4,8,17,21,44,45]. We have shown that transfers can be predicted on the basis of differential productivity, which depends on the importance of learning, in interaction with kinship.

The connection between the life-history of economic production and the transfers observed here provides concrete support for theories emphasizing the importance of skill development for the evolution of animal life histories and breeding systems more generally [45–49]. These insights are reinforced by associations between the skill requirements of food acquisition and transfers of food from adults to offspring among non-human primates [50–54]. Comparative tests are needed to evaluate the importance of learning in fostering the co-occurrence of ‘slow’ life histories and high levels of investment in offspring from parents and other kin (i.e. cooperative breeding), relative to other factors, such as the importance of limited and defensible resources (e.g. territories, dens or burrows) [55,56].

The theory developed here can be interpreted as a generalization of classic parental investment theory [57–59]. The results of the analysis support this generalization by showing that Tsimane’ invest in their grandchildren, siblings, nieces and nephews in ways that take into account their life-stage and relative caloric need. The strength of these effects declines with decreasing genetic relatedness, as predicted by the theory.

While net downward transfers predominate within Tsimane extended families, net upward transfers are observed across a small number of relationship types: from adult children to mothers over age 70; from children-in-law to mothers-in-law, particularly over age 60; from sons in their early 20s to parents; and from daughters-in-law in their early 30s to parents-in-law. Importantly, the between-family analysis reveals that transfers are not unconditional, but rather depend crucially on the relative need of nuclear units within the extended family. Older parents who still support a large number of dependents, for example, are estimated to receive net contributions from their less needy, more productive adult children. On the whole, however, since younger families (with parents under age 30) tend to have relatively lower productivity and higher dependent need (figure 1*b*), resources on average tend to flow from older to younger nuclear families.

The current empirical study focuses entirely on the redistribution of subsistence foods from primary producers to their consumers. As such, contributions to a family's economic/nutritional well-being outside primary production, such as food processing and breastfeeding, are not represented. Similarly structured studies that account for mothers' contributions to children through breastfeeding are needed to complement the measures of transfers of primary production reported in electronic supplementary material, tables E1 and E2. Accounting for effort towards food processing and other complementary roles (as in [7,60]) is similarly worthwhile, but entails its own challenges. In addition to immediate levels of effort and performance, it is important to take into account previous investments in embodied capital necessary to successfully complete each task. For example, if a surgeon has to both transplant a heart and ensure that her children are cared for, a babysitter cannot take equal credit for the heart transplant, although both may have proximately invested roughly equal amounts of time/energy to achieve the joint goal.

The evolutionary and ecological theory for the structure of intergenerational transfers introduced here produces tractable predictions for variation in other populations. While the form of the production curves across small-scale human groups is remarkably general [8,9,17–20,60], shifts in the timing of skill acquisition, physical ability and returns to consumption should be associated with variation in the direction and volume of transfers through specific phases of life. The economic productivity of children and adolescents, for example, has been shown to vary as a function of ecology, owing to differences in predation risk, gains to learning, availability of easy-to-acquire resources, and ability to contribute labour to cultivation [60–62].

The life cycle of aggregate net production within nuclear families may also differ according to socioecology. Data on the caloric productivity of Ache nuclear families [9], for example, suggest a different pattern from that of the Tsimane’ shown in figure 1*b*. On average, young Ache families appear to produce net economic surpluses, whereas older families (with parents in their 40s and 50s) appear to run net economic deficits. This may be attributable to lower early-age fertility and/or lower old-age productivity among the Ache compared to the Tsimane’. The timing, direction and volume of intergenerational transfers between families may be expected to differ as a result.

## Supplementary Material

Electronic Supplementary Material (ESM)

## Data accessibility

The data used in this analysis have been anonymized and made publicly available through the Dryad data repository at http://datadryad.org/review?doi=doi:10.5061/dryad.6m98m.
