# Supplementary material for: Inclusive fitness and differential productivity across the life course determine intergenerational transfers in a small-scale human society
Source: Proc Biol Sci. 2015 Mar 22;282(1803):20142808. doi: 10.1098/rspb.2014.2808 (PMC4345452; doi:10.1098/rspb.2014.2808)
Supplement: Electronic Supplementary Material (ESM) [file rspb20142808supp1.pdf]

Inclusive fitness and differential productivity across the life course determine intergenerational transfers in a small-scale human society:

ELECTRONIC SUPPLEMENTARY MATERIALS (ESM)

Paul L. Hooper, Michael Gurven,
Jeffrey Winking, and Hillard S. Kaplan

January 14, 2015

S1 Details on data collection and preparation

S1.1 Field setting

The Tsimane' are an Amerindian group native to the Beni Department of lowland Bolivia [1, 2]. Tsimane families reside together in residential clusters within villages, most often with close kin, and exhibit relatively high rates of mobility between communities. The Tsimane' subsistence economy consists predominantly of hunting, fishing, and swidden horticulture. Cultivation of rice, manioc, plantains, and maize is labor intensive, with yearly clearing of new fields, and is more limited by inputs of labor than availability of land [3, 4].

The sample utilized in this study comprises 239 nuclear families (married adults or single parents and their immediate dependents) in eight villages ranging in size from 45 to 400 individuals. Trained Tsimane' research assistants resided in each village for an average of 14 months ($\pm$7 SD) and conducted interviews in the Tsimane' language.

S1.2 Production-and-sharing interviews

In six villages, production-and-sharing interview sampling was complete, with all families participating in the study; in two villages, sampling covered a majority ($\geq$63%) of the total community constituting the natural core of the village. One family initially declined to participate, but then joined the study mid-way through the sampling period. Each family was interviewed an average of 46 ($\pm$20 SD) times, yielding a mean of 93 ($\pm$40 SD) sample days per individual.

Production-and-sharing interviews were conducted with adult heads of household (mothers and fathers), or a family member over age 13 if the heads of household were away, in consultation with all others present. Given that food procurement decisions, returns, and consumption are a dominant topic of conversation and communication in Tsimane' families, family members were typically able to provide specific information on the subsistence behavior of other members of the household. Interviews were regularly reviewed and revised by the authors and other North American team members in consultation with local research assistants.

S1.3 Food production and consumption

Daily caloric production rates of game, fish, and horticultural products were estimated for each individual based on the production-and-sharing and field interview datasets. The caloric value of products was derived from Latin American nutritional tables [5, 6]. Credit for game and fish that were acquired cooperatively was divided evenly between acquirers. Credit for horticultural products was divided

between harvesters and those contributing labor to preparing and maintaining the fields from which products were harvested. The relative weight of credit to each laborer was assigned in proportion to the hours of labor they contributed, multiplied by the expected age- and sex-specific efficiency of labor.

To reduce potential biases resulting from uneven sampling across seasons, production rates were calculated separately for each month, then averaged with equal weighting across months; individuals and families with insufficient sampling during the critical harvest months (February–May) were excluded from the analysis, as described in Section S2. Given the present focus on subsistence production, cash-cropped horticultural goods were not included in the tallies. (Futher methodological details are given in Ref. [3].)

Daily consumption requirements (in cals/day) were estimated on the basis of age, sex, and weight according to FAO formulae [7]. Net production (in cals/day) was calculated by subtracting consumption from gross production, while net need (equal to negative net production) was calculated by subtracting gross production from consumption.

S1.4 Transfers

Caloric transfers were calculated from the redistribution of food products recorded in the production-and-sharing dataset. Donor credit for transfers was attributed to primary producers according to the methods described above. The share received by gift recipients was assigned according to the weight of gifts recorded in the interviews; once gift quantities were subtracted, the product's remaining calories were divided among meal recipients in proportion to estimated consumption requirements.

For each type of food t (game, fish, or horticulture), mean gross calories transferred from individual i to individual j per day were calculated in two steps. First, the fraction of i's production of t received by j was calculated by dividing the raw total of calories of t transferred from i to j by the raw total of calories of t transferred from i to all recipients (including i and j). This fraction was then multiplied by the measured daily production rate of t by i, to yield mean gross calories of t transferred per day. (More formally: the gross amount transferred from i to j for each food type t is $G_{ijt} = p_{it} g_{ijt} / \sum_k g_{ikt}$, where p_{ijt} is i's daily production rate for that type, g_{ijt} is the raw total of calories of type t transferred from i to j, and $\sum_k g_{ikt}$ is the raw total of calories of type t transferred from i to all recipients.) This method ensures that transfers reflect an individual's total productivity, as well as specific patterns of sharing for each food type. Calories transferred were summed across food types to yield gross total calories transferred per day.

Net transfers from i to j were calculated as gross transfers from i to j minus

gross transfers from j to i. Net transfers from nuclear family i to nuclear family j were calculated by summing the net tranfers from each member of family i to each member of family j.

S1.5 Kinship

Consanguineous (e.g. parent, sibling) and affinal (e.g. sibling's spouse, spouse's parent) kinship categories and genetic relatedness (r) were calculated between individuals on the basis of ≥ 3 generations of genealogy derived from census and demographic interviews [2]. Mean relatedness between nuclear families i and j was calculated as the mean relatedness of each member of family i to each member of family j.

S2 Details on the statistical analysis

S2.1 Transfers between individuals

1,047 of 1,254 individuals in the full sample had sufficiently detailed information on the production of seasonal horticultural goods to be included in the individual-level analysis. Outliers with net transfers over 7 SDs from the mean were excluded from the analysis (which excluded one observation from the 'parents $\rightarrow$ child' model, and one observation from the 'grandparents $\rightarrow$ grandchild' model), an action which had no effect on the direction or significance of results.

In the individual-level analysis (Tables E1–E7 and Fig. 2), two models were estimated for each type of relationship: first, a sex- and age-stratified model, with 10-year age categories for focal donors (parents, grandparents, spouses, and parents-in-law) and 4-year age categories for focal recipients (children, grandchildren, and children-in-law); and second, an all-ages model stratified by sex.

Random effects for community identity were included in the individual-level models of net transfers in order to capture heterogeneity in transfers across the study communities. The standard deviation and significance of these random effects are reported in Table E8.

The statistical significance of estimated values was bootstrapped by comparing observed values against the values produced from an ensemble of randomly resampled 'null datasets'. To construct the ensemble of null datasets, the sum of net transfers between focal individuals and different categories of kin was recomputed after randomly reshuffling the net transfer values across all individual-individual dyads in each category (e.g. parents and children). The reported p-values represent the fraction of null datasets yielding estimated values $\geq$ observed values.

S2.2 Transfers between nuclear families

The family-level analyses (Tables 1 and 2) were motivated by the fact that, due to regular pooling of food within nuclear families, the amount that family members give to and receive from others is expected to be determined not so much by their own hunger as by the hunger of their families. Thus the effects of need should be plainly observable at the level of the nuclear family. The individual- and family-level analyses in this way are complementary, providing insight into both distinct and overlapping aspects of the sharing system across two scales.

194 of 239 nuclear families in the full sample had sufficiently detailed information on the production of seasonal horticultural goods to be included in the family-level analysis. Of the 194 nuclear families included in the family-level analysis, 175 (90%) contained only biological children of the reproductive-age adults, or no children at all, while 19 included one or more "adopted" dependents. Of the 32 "adoptees" in these families, 19 were grandchildren, 2 were younger siblings, and 11 were more distant kin or non-kin. For this analysis, individuals that changed family membership during data collection due to marriage, divorce, or migration were assigned membership in the family where they resided for the majority of the sample period.

Two types of family-level models were estimated: the first estimated the relationship between transfers and interactions between mean genetic relatedness (r) and the net need of each family (Table 1); the second examined differences in patterns of transfers according to need across four categories of relationship: parent-offspring family pairs, sibling-sibling family pairs, other genetically related family pairs, and unrelated family pairs (Table 2).

Two different but related variables of net caloric need (consumption minus production) were used in the family-level analysis. First, a family's *estimated net need* was calculated on the basis of its demographic composition and population mean age- and sex-specific rates of consumption and production. This can be interpreted as an instrumental variable representing the sum of the expected net need of all members of a nuclear family. Second, *measured net need* was calculated by summing the individual-specific, measured consumption minus production rates of each family member.

Estimated net need was utilized in addition to measured net need because of the possibility of correlated error between measured net need and transfers, since both are calculated using individual-specific production rates. Estimated net need thus allows an evaluation of the effects of need in the absence of this potential source of bias. The two measures may also capture different aspects of the relationship between need and transfers. Estimated need should reflect to a greater extent transfers occuring on the basis of the long-term expected economic and demographic state of families; while measured need should capture to a

greater extent idiosyncratic differences in productivity across families, or vagaries of fortune within the period of sampling. Estimated need is also less affected by individual-level sample/measurement error, since it is based on expectations for age/sex classes.

Net transfer and net need terms were standardized to have mean = 0 and standard deviation = 1. Random effects were included for community identity and the identity of each family, i and j. The standard deviation and significance of these terms are reported in Table E9. The statistical significance of estimates was computed by randomly reshuffling net transfer values across family-family dyads, with p-values representing the fraction of reshuffled null datasets yielding estimates equal to or more extreme than the observed values.

S3 Supplementary tables

Donor	Age	n	Net to children Mean	SE	p
Mother	< 20	24	66.8	316.1	0.194
	20-29	42	516.9	244.9	<0.001
	30-39	46	1042.4	220.7	<0.001
	40-49	30	726.1	275.7	<0.001
	50-59	19	618.8	364.9	0.004
	60-69	10	-391.8	417.6	0.059
	≥ 70	6	-555.6	565.9	0.030
	all	177	559.3	151.4	<0.001
Father	< 20	10	109.0	529.2	0.289
	20-29	39	657.3	251.6	<0.001
	30-39	44	1736.9	232.5	<0.001
	40-49	33	1249.4	251.0	<0.001
	50-59	18	1312.6	387.2	<0.001
	60-69	13	823.1	388.6	0.009
	≥ 70	9	108.4	475.0	0.380
	all	165	1106.8	155.1	<0.001

Table E1: Net transfers (cals/day) from a focal parent to all her/his children as a function of parent age and sex, as estimated by mixed-effect models. These values reflect net transfers of primary food production, and do not include mothers' contributions to infants and young children through breastfeeding. p-values indicate whether the mean deviates significantly from the null expectation of zero.

Recipient	Age	n	Net from parents Mean	SE	p
Daughter	0-3	95	426.9	95.7	0.001
	4-7	76	584.3	97.0	<0.001
	8-11	69	701.4	101.9	<0.001
	12-15	45	446.9	117.6	<0.001
	16-19	34	306.3	129.7	0.001
	20-23	10	317.9	205.9	0.012
	24-27	10	512.5	222.8	0.001
	28-31	16	238.9	192.2	0.052
	32-35	14	78.7	206.3	0.331
	36-39	5	-64.8	248.3	0.363
	<40	374	437.2	71.6	<0.001
Son	0-3	96	299.7	95.8	<0.001
	4-7	76	659.2	100.0	<0.001
	8-11	66	791.8	101.3	<0.001
	12-15	71	504.8	103.0	<0.001
	16-19	46	45.2	112.9	0.322
	20-23	23	-278.2	148.9	0.029
	24-27	12	118.6	205.3	0.247
	28-31	13	-96.6	186.8	0.247
	32-35	9	88.9	264.4	0.314
	36-39	9	15	235.0	0.460
	<40	421	392.52	70.8	<0.001

Table E2: Net transfers (cals/day) to a focal child from her/his parents as a function of child age and sex, as estimated by mixed-effect models. These estimates do not include mothers' contributions to infants and young children through breastfeeding. p-values indicate whether the mean deviates significantly from the null expectation of zero.

Donor	Age	n	Net to grandchildren Mean	SE	p
Grandmother	30-39	9	-44.5	158.6	0.141
	40-49	24	175.5	97.6	0.001
	50-59	19	147.3	113.9	0.042
	60-69	7	86.4	135.7	0.284
	≥ 70	5	12.2	177.0	0.464
	all	64	117.0	68.5	0.002
Grandfather	30-39	3	-65.2	298.1	0.076
	40-49	22	205.4	98.3	<0.001
	50-59	15	182.7	129.0	0.017
	60-69	11	337.3	134.7	0.001
	≥ 70	9	203.9	136.1	0.035
	all	60	221.7	69.5	<0.001

Table E3: Net transfers (cals/day) from a focal grandparent to all her/his grandchildren as a function of grandparent age and sex, as estimated by mixed-effect models. p-values indicate whether the mean deviates significantly from the null expectation of zero.

Recipient	Age	n	Net from grandparents Mean	SE	p
	0-3	81	62.1	60.1	0.003
	4-7	63	88.3	59.6	<0.001
	8-11	41	153.4	62.8	<0.001
	12-15	26	88.0	65.0	<0.001
	16-19	18	91.3	68.5	<0.001
Grand-	20-23	5	34.2	92.9	0.125
daughter	24-27	5	32.4	92.8	0.144
	28-31	5	34.6	100.6	0.151
	32-35	6	57.7	99.8	0.078
	36-39	1	248.4	-	-
	<40	251	87.1	55.13	<0.001
	0-3	77	119.1	60.3	<0.001
	4-7	57	85.0	61.0	<0.001
	8-11	46	166.1	61.1	<0.001
	12-15	40	83.6	63.1	0.001
	16-19	29	8.5	63.7	0.374
Grandson	20-23	12	81.8	72.0	0.051
	24-27	3	41.1	110.5	0.220
	28-31	4	46.9	100.3	0.162
	32-35	3	42.8	129.2	0.164
	36-39	1	100.8	-	-
	<40	272	95.0	55.14	<0.001

Table E4: Net transfers (cals/day) to a focal grandchild from her/his grandparents as a function of child age and sex, as estimated by mixed-effect models. p-values indicate whether the mean deviates significantly from the null expectation of zero.

Donor	Age	n	Net to spouse(s) Mean	SE	p
Wife	<20	26	-412.7	133.9	0.005
	20-29	43	-195.6	104.1	0.035
	30-39	43	-308.9	102.9	0.002
	40-49	32	-94.0	120.7	0.227
	50-59	20	-157.3	152.7	0.171
	60-69	8	-724.2	241.4	0.003
	≥ 70	4	-491.6	341.4	0.071
	all	64	-263.5	51.2	<0.001
Husband	<20	13	357.9	189.4	0.069
	20-29	43	356.2	104.1	<0.001
	30-39	46	215.3	100.7	0.025
	40-49	33	267.4	117.1	0.016
	50-59	17	165.4	165.6	0.196
	60-69	13	230.1	189.4	0.122
	≥ 70	7	362.9	258.1	0.070
	all	60	273.45	51.8	<0.001

Table E5: Net transfers (cals/day) from a focal spouse to her/his spouse(s) as a function of the focal spouse's age and sex, as estimated by mixed-effect models. p-values indicate whether the mean deviates significantly from the null expectation of zero.

Donor	Age	n	Net to children-in-law Mean	SE	p
Mother-in-law	30-39	10	-49.9	260.2	0.307
	40-49	23	-41.1	167.9	0.347
	50-59	16	-179.3	209.8	0.204
	60-69	8	-480.9	235.2	0.082
	≥70	6	-608.9	290.4	0.054
	all	63	-205.8	108.1	0.011
Father-in-law	30-39	2	272.4	755.9	0.109
	40-49	21	104.9	167.2	0.136
	50-59	16	-117.2	224.0	0.217
	60-69	10	71.4	234.9	0.349
	≥ 70	8	135.0	246.5	0.261
	all	57	67.2	111.9	0.160

Table E6: Net transfers (cals/day) from a focal parent-in-law to all her/his children-in-law as a function of parent-in-law age and sex, as estimated by mixed-effect models. p-values indicate whether the mean deviates significantly from the null expectation of zero.

Recipient	Age	n	Net from parents-in-law Mean	SE	p
	12-15	3	321.7	385.2	0.074
	16-19	15	74.7	136.3	0.245
Daughter-	20-23	11	161.0	181.7	0.140
in-law	24-27	10	135.1	164.4	0.148
	28-31	10	199.3	164.3	0.066
	32-35	7	-479.0	272.4	0.037
	36-39	3	-30.7	222.5	0.334
	<40	56	83.1	69.6	0.068
	12-15	0	-	-	-
	16-19	5	-8.5	314.5	0.483
Son-	20-23	10	112.5	157.4	0.165
in-law	24-27	8	254.3	172.4	0.011
	28-31	13	-13.8	157.3	0.466
	32-35	9	120.3	192.7	0.196
	36-39	14	-89.1	157.4	0.216
	<40	48	69.4	69.6	0.092

Table E7: Net transfers (cals/day) to a focal child-in-law from her/his parents-in-law as a function of child-in-law age and sex, as estimated by mixed-effect models. p-values indicate whether the mean deviates significantly from the null expectation of zero.

Table	Response variable	Model	Community SD	p
E1	Net parent → children	Age-stratified	133.4	0.071
E1	Net parent → children	All-ages	253.1	0.005
E2	Net parents → child	Age-stratified	176.3	<0.001
E2	Net parents → child	All-ages	171.8	0.003
E3	Net grandparent → grandchildren	Age-stratified	127.4	0.002
E3	Net grandparent → grandchildren	All-ages	118.6	0.004
E4	Net grandparents → grandchild	Age-stratified	163.2	0.003
E4	Net grandparents → grandchild	All-ages	155.3	<0.001
E5	Net spouse → spouse(s)	Age-stratified	0.0	1.000
E5	Net spouse → spouse(s)	All-ages	0.0	1.000
E6	Net parent-in-law → children-in-law	Age-stratified	145.5	0.096
E6	Net parent-in-law → children-in-law	All-ages	133.8	0.108
E7	Net parents-in-law → child-in-law	Age-stratified	145.5	0.096
E7	Net parents-in-law → child-in-law	All-ages	0.0	1.000

Table E8: Standard deviation and significance of random-effect terms for community in the mixed-effect models presented in Tables E1–E7.

Table	Response variable	Model	Community SD	Community p	Family i SD	Family i p	Family j SD	Family j p
1	Net $i \to j$	1. Estimated net need	0.397	0.012	0.071	0.092	0.087	0.040
1	Net $i \to j$	2. Measured net need	0.399	0.017	0.068	0.131	0.084	0.057
2	Net $i \to j$	1. Mean-only	238.432	0.027	32.465	0.175	38.691	0.115
2	Net $i \to j$	2. Estimated net need	0.441	0.021	0.056	0.269	0.086	0.088
2	Net $i \to j$	3. Measured net need	0.479	0.022	0.000	1.000	0.008	0.081

Table E9: Standard deviation and significance of random-effect terms for community, donor famliy identity i, and recipient family identity j in the mixed-effect models presented in Tables 1 and 2.

References

[1] E. Nordenskiöld, *Forskningar och äventyr i Sydamerika.* Stockholm: Albert Bonniers Förlag, 1915.

[2] M. Gurven, H. Kaplan, and A. Zelada Supa, "Mortality experience of Tsimane Amerindians of Bolivia: Regional variation and temporal trends," *American Journal of Human Biology*, vol. 19, no. 3, pp. 376–398, 2007.

[3] P. L. Hooper, *The structure of energy production and redistribution among Tsimane´ forager-horticulturalists.* PhD thesis, University of New Mexico, 2011.

[4] M. Gurven, M. Borgerhoff Mulder, P. L. Hooper, H. Kaplan, R. Quinlan, R. Sear, E. Schniter, C. Von Rueden, S. Bowles, T. Hertz, and A. Bell, "Domestication alone does not lead to inequality," *Current Anthropology*, vol. 51, no. 1, pp. 49–64, 2010.

[5] FAO/LATINFOODS, *Tabla de Composición de Alimentos de América Latina.* 2009.

[6] U. de São Paulo. Faculdade de Ciências Farmacêuticas. Departamento de Alimentos e Nutrição Experimental/BRASILFOODS, *Tabela Brasileira de Composição de Alimentos-USP. Versão 5.0.* São Paulo: 1998.

[7] FAO, *Human Energy Requirements: Report of a Joint FAO/WHO/UNU Expert Consultation.* Rome: 2001.
